# Insulin Resistance Emerges Early After Glucocorticoid Treatment in Adult Patients With 21‐Hydroxylase Deficiency

**DOI:** 10.1111/1753-0407.70258

**Published:** 2026-07-30

**Authors:** Chenchen Dong, Wencui Wang, Sichang Zheng, Lingxin Deng, Rulai Han, Weiqing Wang, Guang Ning, Shouyue Sun, Lei Ye

**Affiliations:** ^1^ Department of Endocrine and Metabolic Diseases, Shanghai Institute of Endocrine and Metabolic Diseases, Ruijin Hospital Shanghai Jiao Tong University School of Medicine Shanghai China; ^2^ Shanghai National Clinical Research Center for Metabolic Diseases, Key Laboratory for Endocrine and Metabolic Diseases of the National Health Commission of the PR China, Shanghai Key Laboratory for Endocrine Tumor, State Key Laboratory of Medical Genomics, Ruijin Hospital Shanghai Jiao Tong University School of Medicine Shanghai China

**Keywords:** 21‐hydroxylase deficiency, glucocorticoid, insulin resistance

## Abstract

**Background:**

Metabolic disorders, particularly insulin resistance (IR), represent important complications in adults with 21‐hydroxylase deficiency (21OHD). Glucocorticoid (GC) therapy is a known risk factor, yet evidence from longitudinal analyses remains scarce.

**Methods:**

In this study, based on a large, genetically characterized, single‐center cohort of Chinese adults with 21OHD, we performed both cross‐sectional and longitudinal analyses to investigate the risk factors for IR.

**Results:**

We found that nearly one‐third of young adult 21OHD patients had IR. Current GC use remained independently associated with IR (OR 13.30, 95% CI 2.54–69.55; *p* = 0.002), whereas neither genotype nor androgen levels showed an association. We followed 52 patients without IR at baseline; incident IR occurred in 57.1% (4/7) of GC‐naive patients and 68.9% (31/45) of previously GC‐exposed patients, with median times to IR onset of 14.7 and 13.1 months, respectively. Importantly, dexamethasone use was independently associated with incident IR (HR 7.04, 95% CI 1.81–27.34; *p* = 0.005). Daily 1000 mg metformin therapy for 6 months did not significantly improve IR (median HOMA‐IR, 2.50–2.82; *p* = 0.460) and only provided a modest benefit in body weight (median BMI, 23.6–22.5 kg/m^2^; *p* = 0.046).

**Conclusion:**

These findings suggest that ongoing GC therapy, particularly dexamethasone, is a risk factor for IR in adults with 21OHD, and new onset IR generally emerged within approximately 1 year after regular treatment.

## Highlights

Ongoing glucocorticoid therapy, particularly dexamethasone, was independently associated with insulin resistance in adults with 21‐hydroxylase deficiency. Incident insulin resistance generally emerged within 13–15 months after regular treatment exposure. Metformin 1000 mg/day for 6 months modestly reduced BMI but did not significantly improve HOMA‐IR.

## Graphical abstract

In adults with 21‐hydroxylase deficiency, ongoing glucocorticoid therapy, especially dexamethasone, was the main risk factor for insulin resistance. New‐onset insulin resistance emerged early, highlighting the need for metabolic monitoring within the first 1–2 years of treatment.

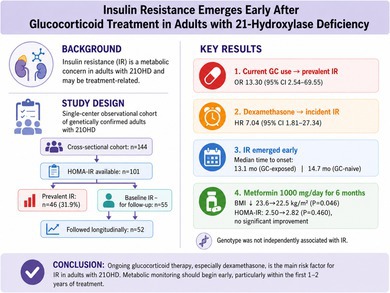

## 1 Introduction

21‐Hydroxylase deficiency (21OHD), the most common form of congenital adrenal hyperplasia (CAH), requires long‐term and often lifelong glucocorticoid (GC) therapy [1, 2]. With improved survival and continuity of care, the clinical focus in 21OHD has gradually expanded from hormonal control to long‐term health outcomes in adulthood, especially metabolic complications such as obesity, insulin resistance (IR), and cardiovascular risk [3, 4, 5].

IR is a central pathophysiological feature underlying a wide range of metabolic disorders and an important early marker of future cardiometabolic risk. In this context, IR has become a clinically relevant concern in patients with 21OHD. Previous studies have shown that patients with CAH have a higher risk of IR than the general population, with reported odds ratios of 5.2 in children and 6.0 in adults [6]. Our group also previously found that patients with CAH had significantly higher fasting insulin levels and homeostasis model assessment of insulin resistance (HOMA‐IR) than controls (1.81 vs. 1.24) [7]. In addition, evidence from hyperinsulinemic–euglycemic clamp studies, the gold standard for assessing insulin sensitivity, further supports the presence of reduced insulin sensitivity in 21OHD, including in non‐obese patients [8, 9].

The mechanisms of IR in adult 21OHD are likely multifactorial. On the one hand, chronic or supraphysiologic GC exposure may impair insulin sensitivity and promote metabolic dysfunction [4, 10]. Several studies have suggested that GC treatment, particularly dexamethasone, may be associated with worse insulin sensitivity [8, 11, 12]. In a longitudinal study of 60 patients, HOMA‐IR increased significantly from baseline to the last follow‐up after prolonged dexamethasone treatment (2.5 ± 1.3 vs. 2.8 ± 1.7, *p* = 0.03) [11]. In a cross‐sectional study including 196 patients, dexamethasone treatment was also associated with higher HOMA‐IR compared with other GC regimens [12]. On the other hand, persistent adrenal androgen excess may also contribute to IR and metabolic dysfunction [13, 14]. Therefore, IR in adult 21OHD may reflect the combined effects of treatment intensity and disease control, rather than a single mechanism.

However, several important clinical questions remain unresolved. In routine practice, it is still unclear which patients are more likely to develop IR during long‐term treatment; whether genotype severity is associated with IR, directly or indirectly through treatment burden; when IR should be monitored more closely, and which treatment strategies may help improve metabolic outcomes. Existing studies are often cross‐sectional and have not clearly separated the respective contributions of genotype severity, hyperandrogenic burden, and current GC exposure [8, 11, 12, 15].

Therefore, based on a large genetically characterized single‐center cohort of Chinese adults with 21OHD, we performed both cross‐sectional and longitudinal analyses to investigate the risk factors of IR. In particular, we aimed to determine whether IR in adult 21OHD is primarily associated with genotype severity, androgen excess, or current GC treatment, and to assess regimen‐specific metabolic risk over time.

## 2 Materials and Methods

### 2.1 Study Design and Participants

**FIGURE 1:**
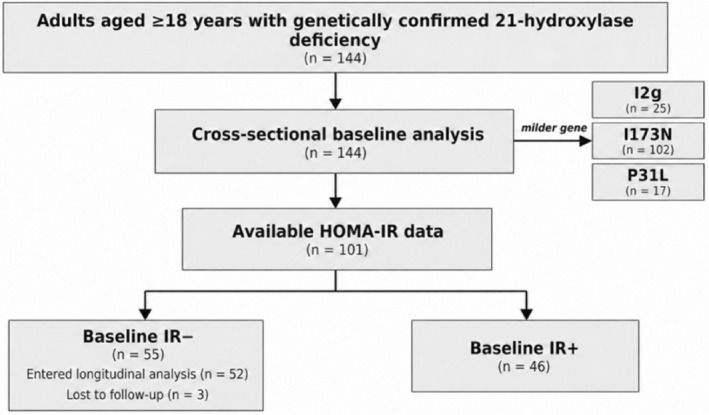
Study flow diagram. IR, insulin resistance.

This was a single‐center observational cohort study including adults (≥ 18 years) with genetically confirmed 21OHD. A total of 144 patients were included in the cross‐sectional analysis. Among them, 101 patients with available fasting glucose and insulin were assessed for IR. Patients without IR at baseline (*n* = 55) were included in longitudinal analysis, of whom 52 had follow‐up data. The study flow is shown in Figure 1.

IR was assessed using HOMA‐IR, calculated as fasting insulin × fasting glucose/22.5. IR was defined as HOMA‐IR > 2.5, based on commonly used thresholds in clinical studies [16, 17, 18]. Biochemical measurements included glucose/lipid profile, adrenal/gonadal hormones. Blood samples were collected in the morning after overnight fasting.

### 2.2 Statistical Analysis

Continuous variables are presented as median (interquartile range) and categorical variables as frequency (percentage). Baseline comparisons among the three groups were performed using the Kruskal–Wallis test for continuous variables and the chi‐squared test for categorical variables. Univariate comparisons between the IR+ and IR− groups were performed using the Mann–Whitney *U* test for continuous variables and Fisher's exact test for categorical variables.

Multivariable analysis was conducted using logistic regression models with stepwise inclusion of covariates to identify independent risk factors for IR. Longitudinal analysis was performed using Cox proportional hazards regression models with time‐varying covariates, with new‐onset IR as the event of interest. Comparisons of BMI and HOMA‐IR before and after metformin intervention were performed using the Wilcoxon signed‐rank test. A two‐sided *p* < 0.05 was considered statistically significant. Statistical analyses were performed using R software (version 4.3.1) and SPSS 26.0.

## 3 Results

### 3.1 Baseline Characteristics Across Genotype Groups

Among 144 adults with 21OHD, the milder allele distribution was dominated by I173N (102/144, 70.8%), followed by I2g (25/144, 17.4%) and P31L (17/144, 11.8%). The cohort was predominantly female (106/144, 73.6%), with median age of 24 years. The baseline median BMI was 23.4 kg/m^2^; 36.1% were overweight, 6.9% were obese, and 9.0% had hypertension. Overall, insulin resistance (HOMA‐IR > 2.5) was identified in 46 of 144 patients (31.9%) and elevated TyG index in 14.6% patients. 89.6% had ever been exposed to GCs treatment, 49.3% were receiving GCs treatment at enrollment.

Across genotype groups, the proportion of females increased from I2g to I173N to P31L (48.0%, 76.5%, and 94.1%, respectively; *p* = 0.002). Morning (8:00 a.m.) cortisol levels were highest in the P31L group (3.7, 5.7, and 9.2 for I2g, I173N, and P31L, respectively; *p* = 0.048). GC exposure and current GC use were similar among the groups. BMI, blood pressure, prevalence of overweight/obesity, and hypertension, fasting glucose, fasting insulin, HOMA‐IR, TyG index, as well as lipid parameters were also similar across genotype groups (Table 1).

**TABLE 1:** Baseline characteristics in 21OHD patients with different genotypes.

Variable	Total (*N* = 144)	I2g (*n* = 25)	I173N (*n* = 102)	P31L (*n* = 17)	*p*
Demographics and anthropometry					
Age, year^a^	24 (20–30)	22 (18–28)	25 (21–31)	25 (21–28)	0.263
Female sex, *n*/*N* (%)	106/144 (73.6)	12/25 (48.0)	78/102 (76.5)	16/17 (94.1)	**0.002**
Height, cm	155.0 (150.0–160.0)	156.0 (150.0–161.0)	152.5 (149.0–159.8)	158.0 (155.0–160.0)	0.079
Weight, kg	55.0 (49.0–61.0)	55.5 (47.5–63.1)	55.0 (49.1–60.0)	55.7 (55.0–61.0)	0.909
BMI, kg/m^2^	23.4 (21.5–26.2)	23.8 (20.3–26.1)	23.5 (21.5–26.1)	22.0 (21.5–25.1)	0.787
Overweight (BMI ≥ 24), *n*/*N* (%)	52/144 (36.1)	10/25 (40.0)	38/102 (37.3)	4/17 (23.5)	0.500
Obese (BMI ≥ 28), *n*/*N* (%)	10/144 (6.9)	3/25 (12.0)	6/102 (5.9)	1/17 (5.9)	0.550
SBP, mmHg	118 (110–127)	112 (110–120)	119 (110–128)	114 (110–120)	0.293
DBP, mmHg	73 (69–80)	76 (72–80)	73 (70–80)	70 (66–74)	0.173
Hypertension, *n*/*N* (%)	13/144 (9.0)	2/25 (8.0)	10/102 (9.8)	1/17 (5.9)	0.856
Glucocorticoid exposure					
GC‐naive, *n*/*N* (%)^b^	15/144 (10.4)	2/25 (8.0)	10/102 (9.8)	3/17 (17.6)	0.563
GC ever exposed, *n*/*N* (%)^c^	129/144 (89.6)	23/25 (92.0)	92/102 (90.2)	14/17 (82.4)	0.563
Current GC use, *n*/*N* (%)^d^	71/144 (49.3)	12/25 (48.0)	52/102 (51.0)	7/17 (41.2)	0.748
DX, *n*/*N* (%)	28/144 (19.4)	5/25 (20.0)	21/102 (20.6)	4/17 (23.5)	0.562
HC, *n*/*N* (%)	14/144 (9.7)	3/25 (12.0)	10/102 (9.8)	1/17 (5.9)	0.805
PRED, *n*/*N* (%)	10/144 (6.9)	2/25 (8.0)	7/102 (6.9)	1/17 (5.9)	0.964
DX + HC, *n*/*N* (%)	19/144 (13.2)	4/25 (16.0)	14/102 (13.7)	1/17 (5.9)	0.609
Glucose and insulin metabolism					
Fasting glucose, mmol/L	4.87 (4.50–5.13)	4.79 (4.50–5.12)	4.88 (4.46–5.11)	4.95 (4.74–5.20)	0.570
Fasting insulin, mU/L	9.4 (7.0–15.3)	10.4 (9.1–15.6)	8.9 (6.7–15.0)	9.6 (7.1–15.3)	0.532
HOMA‐IR	2.08 (1.41–3.29)	2.13 (1.80–2.91)	2.07 (1.35–3.34)	2.04 (1.41–3.32)	0.889
Insulin resistance (HOMA‐IR > 2.5), *n*/*N* (%)	46/144 (31.9)	11/25 (44.0)	30/102 (29.4)	5/17 (29.4)	0.364
TyG index	8.10 (7.77–8.50)	7.98 (7.74–8.32)	8.19 (7.81–8.50)	8.38 (7.70–8.81)	0.615
Elevated TyG (≥ 8.5), *n*/*N* (%)	21/144 (14.6)	3/25 (12.0)	15/102 (14.7)	3/17 (17.6)	0.877
IFG (FPG ≥ 5.6 mmol/L), *n*/*N* (%)	8/144 (5.6)	2/25 (8.0)	5/102 (4.9)	1/17 (5.9)	0.831
Lipid profile					
TG, mmol/L	0.95 (0.64–1.25)	0.84 (0.61–1.08)	0.97 (0.66–1.24)	0.81 (0.54–1.70)	0.608
TC, mmol/L	3.84 (3.27–4.77)	4.09 (3.46–4.72)	3.79 (3.25–4.71)	4.24 (3.14–4.78)	0.693
HDL‐C, mmol/L	1.18 (0.98–1.36)	1.23 (1.07–1.35)	1.15 (0.96–1.35)	1.25 (0.98–1.42)	0.503
LDL‐C, mmol/L	2.36 (1.89–3.04)	2.45 (2.08–3.15)	2.32 (1.88–2.84)	2.33 (1.94–3.09)	0.716
Adrenal and gonadal hormones					
17‐OHP, ng/mL	37.0 (2.2–38.0)	37.0 (11.9–44.0)	37.0 (1.3–38.0)	33.5 (10.0–37.5)	0.458
AD, ng/mL	6.80 (1.60–10.00)	7.58 (1.94–10.00)	6.46 (1.17–10.25)	6.68 (1.56–10.00)	0.927
Progesterone, ng/mL	5.69 (1.81–10.85)	5.69 (2.64–10.85)	7.15 (1.81–15.08)	3.10 (1.41–4.82)	0.041
Testosterone, ng/ml	1.65 (0.33–3.79)	1.90 (0.47–5.66)	1.79 (0.23–3.96)	1.18 (0.52–1.68)	0.231
DHEAS, μg/dL	117.0 (18.3–373.6)	205.8 (28.1–377.5)	107.5 (16.6–352.7)	252.7 (39.6–514.2)	0.522
ACTH, pg/mL	120.4 (27.3–244.3)	168.4 (65.7–407.4)	126.0 (22.2–263.2)	53.8 (35.9–112.4)	0.101
Cortisol (8 a.m.), μg/dL	5.5 (1.0–8.7)	3.7 (1.7–6.5)	5.7 (0.8–8.1)	9.2 (4.8–12.6)	0.048
SHBG, nmol/L	33.4 (21.40–48.7)	30.8 (20.2–39.5)	35.5 (24.2–52.4)	32.7 (26.5–35.2)	0.358
LH, mIU/mL	3.01 (1.20–4.97)	1.93 (0.19–4.17)	2.98 (1.21–4.99)	4.46 (2.27–5.64)	0.173
FSH, mIU/mL	4.48 (2.44–6.32)	3.90 (0.58–5.84)	4.39 (2.45–6.32)	5.34 (4.15–6.96)	0.206

*Note:* Bold values indicate statistically significant differences (*p* < 0.05).

Abbreviations: 17‐OHP, 17‐hydroxyprogesterone; ACTH, adrenocorticotropic hormone; AD, androstenedione; BMI, body mass index; DBP, diastolic blood pressure; DHEAS, dehydroepiandrosterone sulfate; DX, dexamethasone; FPG, fasting plasma glucose; FSH, follicle‐stimulating hormone; GC, glucocorticoid; HC, hydrocortisone; HDL‐C, high‐density lipoprotein cholesterol; HOMA‐IR, homeostasis model assessment of insulin resistance; IFG, impaired fasting glucose; LDL‐C, low‐density lipoprotein cholesterol; LH, luteinizing hormone; PRED, prednisone/prednisolone; SBP, systolic blood pressure; SHBG, sex hormone‐binding globulin; TC, total cholesterol; TG, triglycerides; TyG, triglyceride‐glucose index.

^a^ Age was defined as age at study enrollment.

^b^ GC‐naive defined as never treated with glucocorticoids before baseline.

^c^ GC ever exposed: any history of GC use before baseline.

^d^ Current GC use was defined as ongoing glucocorticoid treatment at study enrollment.

### 3.2 Cross‐Sectional Associations With IR

In the cross‐sectional analysis, patients were stratified by IR status. Patients with and without IR were similar in age, sex, height, blood pressure, and genotype distribution. Weight tended to be higher in the insulin‐resistant group (56.0 vs. 54.8 kg, *p* = 0.055), whereas BMI was significantly higher (24.2 vs. 22.9 kg/m^2^, *p* = 0.013).

Compared with patients without IR, those with IR were more likely to be receiving GC therapy, particularly dexamethasone, at the time of study entry (73.9% vs. 30.9% and 47.8% vs. 10.9%, respectively; both *p* < 0.001). Prior GC exposure, however, was common overall and did not differ between groups.

**FIGURE 2:**
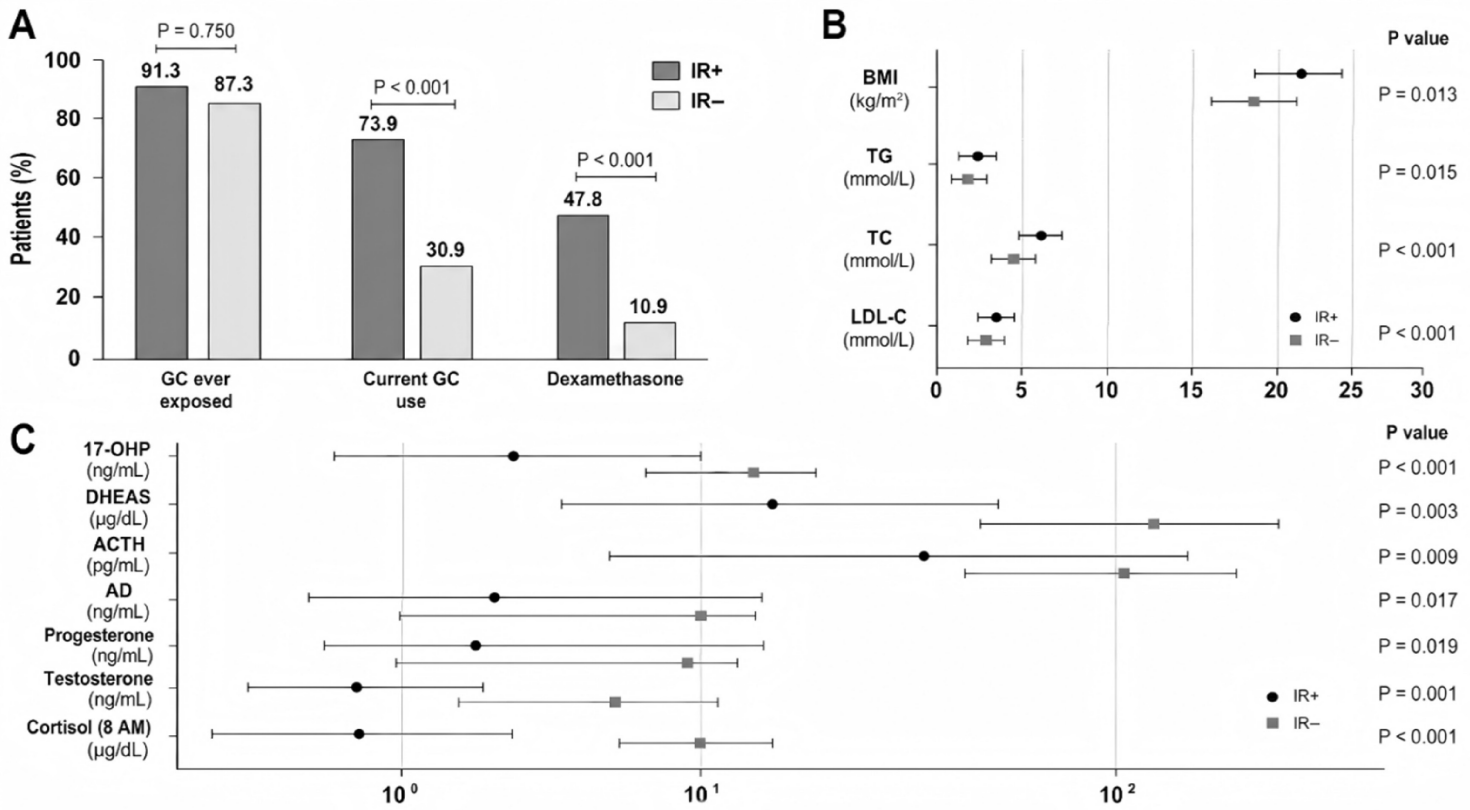
Univariate correlates of baseline insulin resistance. (A) Comparison of glucocorticoid exposure and dexamethasone use between patients with and without insulin resistance. (B) Comparison of BMI and lipid parameters between patients with and without insulin resistance. (C) Comparison of adrenal and gonadal hormone levels between patients with and without insulin resistance. 17‐OHP, 17‐hydroxyprogesterone; ACTH, adrenocorticotropic hormone; AD, androstenedione; BMI, body mass index; CI, confidence interval; GC, glucocorticoid; OR, odds ratio; TC, total cholesterol; TG, triglycerides.

Patients with IR had higher fasting glucose, fasting insulin, HOMA‐IR, and TyG index (all *p* < 0.001). Differences were also observed in lipid profile, with higher triglycerides (*p* = 0.015), total cholesterol (*p* < 0.001), and LDL‐cholesterol (*p* < 0.001) among patients with IR. Compared with patients without IR, those with IR had lower 17‐OHP, ACTH, AD, DHEAS, progesterone, testosterone, SHBG, and morning cortisol (all *p* < 0.05). These univariate associations are summarized in Figure 2.

### 3.3 Multivariable Determinants of Baseline IR

In multivariable logistic regression analysis, genotype was not independently associated with baseline IR in any of the three models. Using I173N as the reference, neither I2g nor P31L was significantly associated with IR after adjustment for age, sex, and BMI in Model 1, and this remained unchanged after further adjustment for current GC use in Model 2 and for triglycerides and testosterone in Model 3. Age and sex were also not independently associated with IR.

In Model 1, BMI was independently associated with baseline IR (OR 1.21, 95% CI 1.05–1.39; *p* = 0.008). After inclusion of current GC use in Model 2, BMI remained significantly associated with IR (OR 1.23, 95% CI 1.03–1.45; *p* = 0.019), and current GC use was independently associated with higher odds of IR (OR 12.35, 95% CI 3.72–40.97; *p* < 0.001). In Model 3, after further adjustment for triglycerides and testosterone, current GC use remained independently associated with IR (OR 13.30, 95% CI 2.54–69.55; *p* = 0.002), and triglycerides were also independently associated with IR (OR 10.76, 95% CI 2.30–50.46; *p* = 0.003), whereas testosterone and BMI were not statistically significant (Table 2).

**TABLE 2:** Multivariable analysis of factors associated with insulin resistance.

Variable	Model 1 (genotype + demographics)		Model 2 (+ current GC use)		Model 3 (+ TG + testosterone)	
	OR (95% CI)	*p*	OR (95% CI)	*p*	OR (95% CI)	*p*
I2g (vs. I173N)	0.60 (0.18–2.01)	0.404	0.66 (0.18–2.49)	0.540	0.48 (0.11–2.15)	0.339
P31L (vs. I173N)	1.31 (0.30–5.83)	0.721	2.49 (0.38–16.39)	0.342	0.84 (0.08–9.01)	0.882
Age, year	0.98 (0.94–1.03)	0.525	1.01 (0.95–1.08)	0.710	0.96 (0.88–1.05)	0.356
Female sex	0.92 (0.31–2.71)	0.885	0.47 (0.13–1.68)	0.243	0.28 (0.06–1.30)	0.103
BMI, kg/m^2^	1.21 (1.05–1.39)	**0.008**	1.23 (1.03–1.45)	**0.019**	1.14 (0.95–1.36)	0.170
Current GC use	—		12.35 (3.72–40.97)	**< 0.001**	13.30 (2.54–69.55)	**0.002**
TG, mmol/L	—		—		10.76 (2.30–50.46)	**0.003**
Testosterone, ng/mL	—		—		0.92 (0.69–1.22)	0.564

*Note:* Bold values indicate statistically significant associations (*p* < 0.05).

Abbreviations: CI, confidence interval; GC, glucocorticoid; OR, odds ratio; TG, triglycerides.

### 3.4 Longitudinal Risk of Incident IR

A total of 55 patients showed no IR at baseline. Fifty‐two (94.5%) patients were followed with the median follow‐up duration as 28.2 months and three visits per patient. Incident IR occurred in 4 of 7 GC‐naive patients (57.1%) and 31 of 45 previously GC‐exposed patients (68.9%). The median times to IR onset was 14.7 and 13.1 months, respectively.

In time‐varying Cox proportional hazards models based on treated intervals, dexamethasone use was consistently associated with a higher risk of incident IR (Tables 3A and 3B). Compared with nondexamethasone GCs, dexamethasone use was associated with incident IR in Model 1 (HR 4.87, 95% CI 1.79–13.24; *p* = 0.002), and this association remained significant after adjustment for age, sex, and BMI in Model 2 (HR 5.04, 95% CI 1.51–16.77; *p* = 0.008) and for HC‐equivalent dose in Model 3 (HR 7.04, 95% CI 1.81–27.34; *p* = 0.005).

**TABLE 3A:** Longitudinal analysis of incident insulin resistance. (A) Follow‐up and outcome.

Characteristic	Value
Follow‐up	
Patients with follow‐up, *n*/*N* (%)	52/55 (94.5)
Visits per patient	3 (2–5)
Duration, months	28.2 (12.0–48.0)
Outcome—GC‐naïve (*n* = 7)	
Incident IR, *n* (%)	4/7 (57.1)
Time to IR, months	14.7 (10.0–31.5)
Outcome—GC‐exposed (*n* = 45)	
Incident IR, *n* (%)	31/45 (68.9)
Time to IR, months	13.1 (6.5–47.5)

**TABLE 3B:** Longitudinal analysis of incident insulin resistance. (B) Time‐varying Cox proportional hazards regression (treated intervals).

Variable	Model 1 (GC type)		Model 2 (+ demographics)		Model 3 (+ HC equiv. dose)	
	HR (95% CI)	*p*	HR (95% CI)	*p*	HR (95% CI)	*p*
DX (vs. non‐DX GC)	4.87 (1.79–13.24)	0.002	5.04 (1.51–16.77)	0.008	7.04 (1.81–27.34)	0.005
Age, year	—		1.03 (0.96–1.09)	0.445	1.04 (0.97–1.12)	0.304
Female sex	—		1.12 (0.33–3.82)	0.859	1.05 (0.31–3.64)	0.934
BMI, kg/m^2^	—		0.88 (0.77–1.00)	0.057	0.85 (0.74–0.99)	0.041
HC equiv., per 10 mg/day	—		—		1.14 (0.67–1.93)	0.639

Abbreviations: BMI, body mass index; CI, confidence interval; DX, dexamethasone; GC, glucocorticoid; HC equiv. dose, hydrocortisone‐equivalent dose; HR, hazard ratio; IR, insulin resistance.

In the fully adjusted Model 3, HC‐equivalent dose was not independently associated with incident IR (HR 1.14 per 10 mg/day, 95% CI 0.67–1.93; *p* = 0.639). BMI showed an inverse association with incident IR (HR 0.85, 95% CI 0.74–0.99; *p* = 0.041).

### 3.5 Metformin Treatment in Patients on Dexamethasone

**FIGURE 3:**
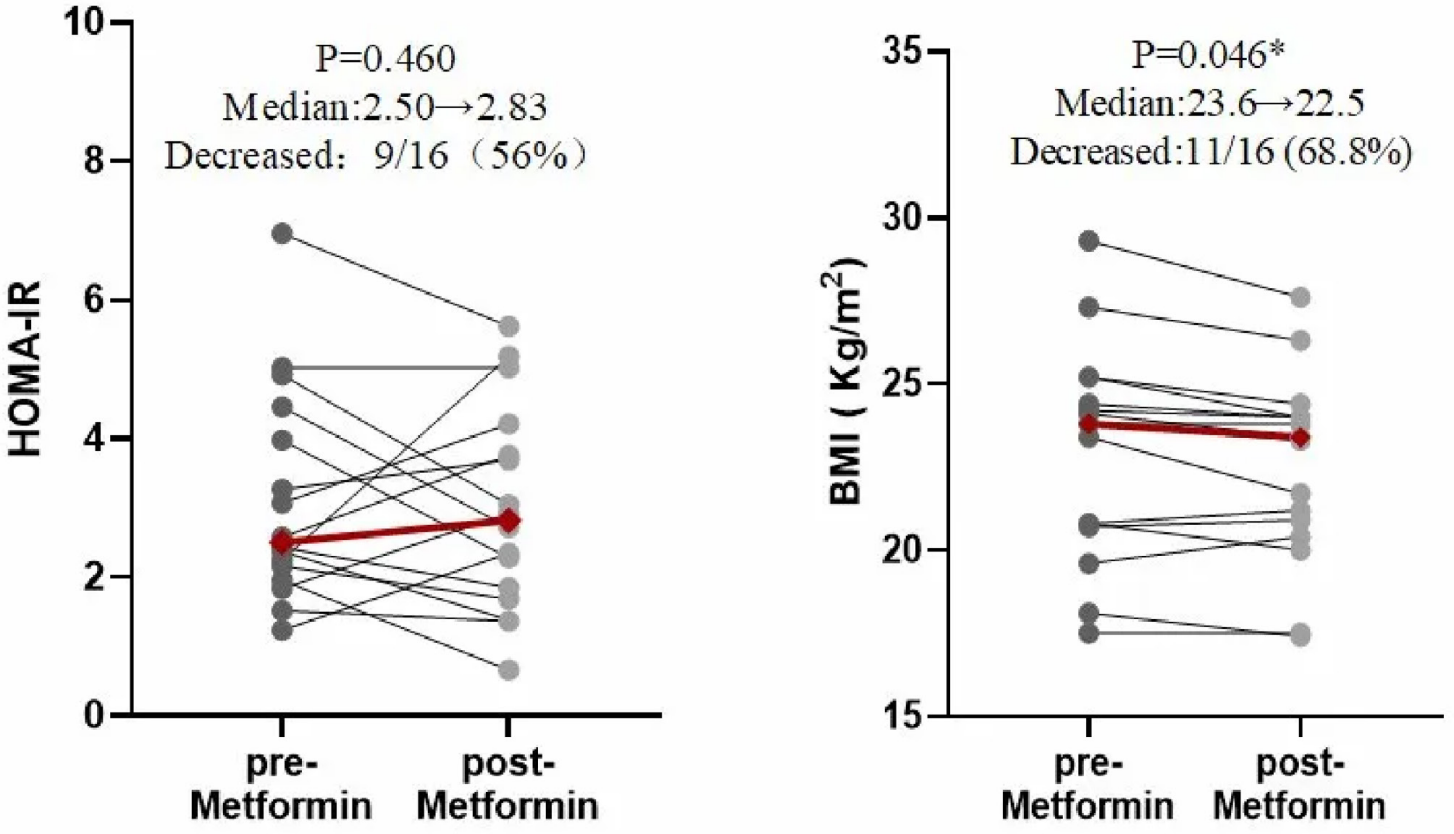
Changes in HOMA‐IR and BMI after metformin treatment. Individual paired values are shown, and the red line indicates the change in median values before and after treatment. BMI, body mass index; HOMA‐IR, homeostasis model assessment of insulin resistance.

In patients receiving an unchanged dexamethasone regimen, pre‐ and posttreatment comparisons after 6 months of metformin therapy (500 mg twice daily) showed a significant decrease in BMI (median, 23.6–22.5 kg/m^2^; *p* = 0.046). Nine of 16 patients showed a reduction of HOMA‐IR, although did not change significantly (median, 2.50–2.82; *p* = 0.460). Eight patients had baseline IR, one returned to normal. Whereas among the eight patients without baseline IR, two developed new IR during follow‐up (Figure 3).

## 4 Discussion

In this study, we found in young adult patients with 21OHD, IR was independently associated with ongoing GC therapy, particularly dexamethasone, with a median time from treatment initiation to onset of 14 months. Moreover, metformin at a daily dose of 1000 mg did not effectively improve IR.

Our results extend previous observations that metabolic abnormalities are frequent in CAH, but also refine the interpretation of risk in adult 21OHD. In a cross‐sectional cohort study reported by Finkielstain et al., which included 244 children and adults with CAH, IR was observed in 27% of children with classic CAH and in 38% and 20% of adults with classic and nonclassic CAH, respectively [19]. And in our cohort of adult patients with 21OHD, IR was present in 46 patients, accounting for 31.9% of the total cohort. Together, these findings indicate that IR is a common metabolic complication in adults with CAH, particularly those with 21OHD. Other earlier studies have also linked CAH to adverse cardiometabolic profiles [20, 21], and some have suggested that dexamethasone‐treated patients may have worse insulin sensitivity than those receiving other regimens [11].

In this context, our study adds two important points: first, dexamethasone exposure is independently associated with incident IR even when hydrocortisone‐equivalent dose was taken into account. Regimen type, rather than dose equivalence alone, is an important determinant of metabolic risk in routine care of adult 21OHD. In the cross‐sectional analysis, patients with baseline IR were substantially more likely to be receiving GC therapy at study entry, particularly dexamethasone. In the time‐varying Cox analysis, dexamethasone exposure remained associated with a markedly higher risk of incident IR than nondexamethasone GCs across treated intervals. Notably, this association persisted after adjustment for hydrocortisone‐equivalent dose. This pattern suggests that the adverse metabolic effect of dexamethasone may not be fully captured by nominal dose conversion alone. Differences in duration of action, tissue exposure, circadian disruption, and cumulative suppression may all contribute. Clinically, these findings support greater caution when long‐acting GCs are used for long‐term control in young adults with 21OHD. Notably, in our patients with IR, lower levels of 17‐OHP, ACTH, DHEAS, testosterone, free testosterone, SHBG, and morning cortisol were observed, excluding undertreatment as a contributing factor.

The second clinically relevant finding is the timing of IR emergence during follow‐up. Among patients free of IR at baseline, incident IR occurred in both treatment‐naïve and prior GC exposed groups, with a median onset time of approximately 13–15 months This suggests that IR in adult 21OHD may develop relatively early after regular treatment exposure. Although the exact onset time should be interpreted cautiously given the interval‐based observational design, our data indicate that the first 1–2 years after treatment initiation or re‐initiation may represent a particularly relevant window for metabolic surveillance. From a practical standpoint, this supports closer early monitoring of glucose‐insulin indices, especially in patients treated with dexamethasone.

The exploratory metformin analysis should also be interpreted in this treatment context. In patients who remained on an unchanged dexamethasone regimen, 6 months of metformin treatment (1000 mg daily) was associated with a modest reduction in BMI but not with a significant overall improvement in HOMA‐IR. However, metformin was found to effectively reverse IR in the 2014 metformin study [13]. The differing findings may have two explanations: first, baseline IR (8.6 vs. 2.5) and BMI (29.2 vs. 23.6) were substantially higher in that study than in our cohort; second, the metformin dose used was 2.5–3.0 g/day compared with 1.0 g/day in our study. The efficacy of metformin in improving IR depends on the degree of baseline resistance—the more severe the resistance, the better the response—and the effect is dose‐dependent. Our results suggest that 1000 mg daily provides limited improvement in IR, and therefore an increased dose of metformin should be considered in managing 21OHD patients with IR.

The absence of a strong independent association between genotype categories and IR in this cohort should be interpreted with caution. This finding does not diminish the well‐established importance of CYP21A2 genotype for diagnosis, phenotypic classification, or early‐life management. Instead, it raises the possibility that once patients enter adult follow‐up, metabolic outcomes may be increasingly influenced by modifiable clinical factors, particularly treatment history and GC regimen, potentially overshadowing genotypic contributions. In our cohort, baseline BMI, blood pressure, glucose indices, lipid parameters, and the prevalence of IR were broadly similar across the I2g, I173N, and P31L groups. This suggests that, at least in this adult cohort and under routine treatment conditions, genotype alone may offer relatively limited added value for metabolic risk stratification compared with treatment‐based assessment. However, we cannot exclude the possibility of modest genotype effects that were not detectable given our sample size.

Our findings have practical implications for adult management of 21OHD. First, ongoing GC exposure, particularly dexamethasone treatment, should be considered an important marker of metabolic risk. Second, because incident IR may emerge within the first 1–2 years of regular treatment, earlier and more systematic metabolic monitoring may be warranted in young adults, including those without baseline IR. Finally, if metformin is used to improve IR, a dose higher than 1.0 g per day should be considered.

Several limitations should also be acknowledged. First, cumulative lifetime GC exposure could not be reconstructed precisely, and this may be relevant to adult metabolic outcomes. Second, the number of longitudinal events and the number of metformin‐treated patients were limited, reducing precision in subgroup analyses. Third, IR was defined using HOMA‐IR rather than clamp‐based metabolic phenotyping. Fourth, because this was an observational study, treatment selection was not random, and residual confounding by disease severity or prescribing patterns cannot be fully excluded.

## 5 Conclusion

In conclusion, in adult 21OHD, IR appears to be more closely associated with ongoing GC treatment, particularly dexamethasone exposure; incident IR generally emerged within approximately 1 year after regular treatment. These findings support closer metabolic surveillance in young adults with 21OHD and suggest that treatment‐aware risk stratification may be considered in adult follow‐up.

## Author Contributions

Chenchen Dong, Wencui Wang, Sichang Zheng: data collection; analysis; original draft. Lingxin Deng, Rulai Han: data analysis. Weiqing Wang, Guang Ning: supervision; resources. Shouyue Sun, Lei Ye: conceptualization; project administration; funding acquisition; writing; review and editing. All authors read and approved the final manuscript.

## Funding

This work was funded by the National Science and Technology Major Project (2023ZD0517900) and the Chinese Academy of Medical Sciences Non‐profit Central Research Institute Fund (2023‐PT320‐05).

## Ethics Statement

The study was approved by the Ethics Committee of Ruijin Hospital (ethical approval No. 2021‐238).

## Consent

Written informed consent was obtained from all participants. The authors confirm that patient consent forms have been obtained for this article.

## Conflicts of Interest

The authors declare no conflicts of interest.

## Data Availability

The data that support the findings of this study are available on request from the corresponding author. The data are not publicly available due to privacy or ethical restrictions.
